# WFDC1 expression identifies memory CD4 T- lymphocytes rendered vulnerable to cell-cell HIV-1 transfer by promoting intercellular adhesive junctions

**DOI:** 10.1186/1742-4690-8-29

**Published:** 2011-05-05

**Authors:** Raymond A Alvarez, Georgina Thorborn, James L Reading, Shalini Kamu Reddy, Annapurna Vyakarnam

**Affiliations:** 1 Department of Infectious Diseases, King's College London, U.K

## Abstract

**Background:**

Elucidating mechanisms that promote HIV-1 transfer between CD4^+ ^T-lymphocytes and their subsequent loss is of importance to HIV-1 pathogenesis. We recently reported that whey acidic protein, ps20, promotes cell-free HIV-1 spread through ICAM-1 modulation. Since ICAM-1 is pivotal in cell conjugation and intercellular HIV-1 transfer, this study examines ps20 effects on HIV-1 spread between T lymphocytes.

**Results:**

We demonstrate intrinsic ps20 variability in primary CD4^+ ^T-lymphocyte clonal populations and a significant positive correlation between endogenous ps20 levels and virus transfer involving fusion resulting in a spreading infection that could be reversed by the addition of reverse transcriptase inhibitors. Blocking anti-ps20 antibody or siRNA mediated ps20 knockdown, significantly reduced virus transfer. Conversely, virus transfer was promoted by ectopic ps20 expression or by exogenous addition of recombinant ps20. A higher frequency of virological synapse formation was evident in cocultures of HIV-1 infected donor T-cells with ps20^high ^v ps20^low/intermediate ^targets. Blocking ps20 inhibited T-lymphocyte conjugate formation and ICAM-1 expression, and was as potent as ICAM-1 in inhibiting HIV-1 transfer.

**Conclusions:**

Therefore ps20 is a novel marker of CD4^+ ^T-cells rendered vulnerable to HIV-1 infection by regulating the fundamental biologic process of intercellular conjugate formation and consequently of potential importance in HIV-1 pathogenesis.

## Background

Understanding the mechanisms by which retroviruses spread from one cell to another is of central importance to disease pathogenesis as this process enables viruses to effectively escape immune responses. Three modes of cell contact have been described which are capable of transmitting retroviruses. One mode is through the formation of filopodial bridges, which are protrusions that originate from uninfected target cells that become tethered to infected donor cells through the surface expression of viral ENV proteins [1]. After tethering, both MLV and HIV-1 were shown to travel along the outside of these bridge structures onto the surface of target cells [1]. A similar mode of retroviral transfer involves thin elongated structures called nanotubes, which form when two T cells come into contact and begin to move apart, independent of virus protein expression and described in HIV-1 transmission [2]. Lastly, a highly prevalent mode of virus transfer, occurs through the close apposition of infected and uninfected cells which form cellular conjugates [3,4] leading to the formation of virological synapses (VS). A VS forms when CD4 and HIV-1 Env and Gag polarize to conjugate interfaces in a microtubule- and actin- dependent manner, allowing for the rapid and direct transfer of virus from infected to uninfected cells [3-10]. A recent study demonstrated conjugate formation preceding and leading to Gag redistribution/polarization with VS formation detected in 80% of conjugates formed [11]. Similarly, the formation of multiple conjugates precedes the formation of multiple VS termed "polysynapses" [12] and is postulated as an efficient mode of virus dissemination *in vivo*, enabling a single infected cell to infect multiple target cells, as observed in the cervix and lymph nodes of SIV^+ ^Macaques [12].

Several host factors beyond the HIV-1 receptor/coreceptor complex can regulate the process of cell-cell HIV-1 transfer depending on whether the conjugates formed are between CD4^+ ^T cells or between CD4^+ ^T cells and dendritic cells. These include adhesion molecules, lipid raft components, signalling molecules and the tetraspanins [6,13-22]. More recently, our laboratory identified a novel HIV-1 enhancing pathway, namely the whey acidic protein, ps20, in memory CD4^+ ^T lymphocytes that promotes cell-free HIV-1 replication through the modulation of ICAM-1 surface expression [23]. Blocking endogenous ps20 suppressed HIV-1 replication, while the exogenous addition of recombinant ps20 promoted infection. Furthermore, blocking anti-ps20 Ab suppressed ICAM-1 surface expression [23]. Cell adhesion antigens like ICAM-1 and integrins (e.g. like LFA-1 and α4β7 [17,18,24-27]), can be exploited by viruses like HIV-1 to promote spreading infection. Specifically, budding cell-free HIV-1 particles that incorporate ICAM-1 bind target cells better through cognate LFA-1 binding [24-27]. Additionally, ICAM-1 can promote cell-to-cell HIV spread by stabilising virus fusion to target cells and VS formation [17,26,27] and anti-ICAM-1 blocking antibody can reduce VS formation by ~30% [17]. Together, these observations prompted us to test the hypothesis that ps20 can promote cell-cell HIV transfer by modulating ICAM-1 expression.

WFDC1/ps20 is a member of the extended whey acidic protein (WAP) family, identified by a highly conserved 4-disulphide core domain, which includes a number of small, secreted proteins found within mucosal secretions [28,29]. Of the 18 human members, only three, namely secretory lymphocyte protease inhibitor (SLPI), Elafin and more recently ps20, have ascribed functions. All three proteins appear multifunctional; SLPI and Elafin possess anti-microbial activity, including anti-HIV-1 activity, as well anti-protease and anti-inflammatory activity [28-30]. Consequently, these proteins are implicated in innate immunity by providing broad anti-microbial cover and by negating the damaging effects of host and pathogen proteases and limiting immune activation [28-30]. To date, ps20 has not been ascribed with anti-microbial activity or anti-protease activity, and in contrast to SLPI and Elafin [30], ps20 promotes HIV-1 infection [23]. A previous study highlighted the ability of ps20 to promote wound healing, cell migration and angiogenesis [31]. All these processes require the modulation of adhesion molecules [32,33], and therefore ps20 function is postulated to involve cell-extracellular matrix or cell-cell interactions [31,34]. In this paper, we provide data in support of this contention by demonstrating that HIV-1 exploits ps20-mediated regulation of the quality and quantity of T lymphocyte-T lymphocyte (T-T) conjugate formation and ICAM-1 expression in the process of cell-cell virus transfer and ps20 to be a novel marker of CD4+ T cells that are highly vulnerable to HIV-1 infection.

## Results

### Jurkat CD4^+ ^T cells stably transduced to express ps20, are rendered more susceptible to T-T HIV-1 transfer

Screening steady state ps20 mRNA in ten primary clones from multiple donors confirmed profound heterogeneity in ps20 levels spanning 5 logs (Additional file 1 figure S1A) and confirmed ps20 expression, in the transduced J-ps20^high ^cells, falls within the range seen in primary clones. As ps20 expression in this panel segregated naturally into three distinct clusters, we arbitrarily assigned populations to be ps20^high ^(RCN above 0.1), ps20^Intermediate ^(ps20^inter^)(RCN 0.001-0.1) and ps20^low ^(RCN below 0.001). Ps20 mRNA expression in J-ps20^high ^cells was 3-logs higher than J-ps20^inter ^cells; accordingly, J-ps20^high ^cultures were clearly ps20 protein positive (Additional file 1 figure S1B). A 23-fold higher level of infection in J-ps20^high ^vs. J-ps20^inter ^cells was noted in a spreading infection assay (Additional file 1 figure S1C). Blocking anti-ps20 Ab reduced single-cycle infection by 2.8-fold in the J-ps20^high ^population (Additional file 1 figure S1D). These data extend previous observation that human ps20 promotes cell-free HIV-1 infection [23].

**Figure 1:**
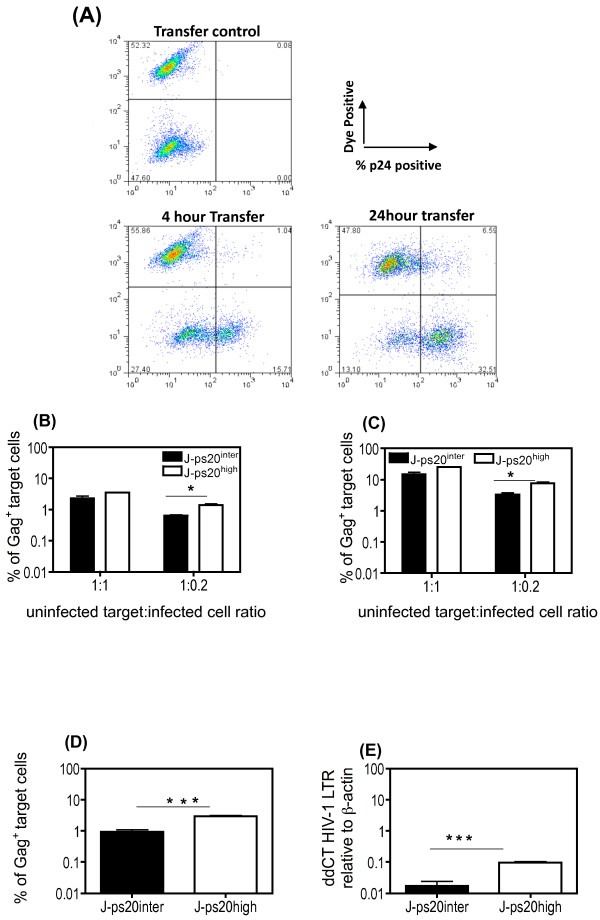
**Jurkat CD4^+ ^T cells stably transduced to express full-length human ps20 are rendered more susceptible to T-T HIV-1 transfer**. (A) Representative dot plots of dye labelled target cells co-cultured with uninfected (transfer control) or infected donor cells at 4 and 24 hours post co-culture. (B) Mean percentage of Gag^+ ^J-ps20^inter ^vs. J-ps20^high ^target cells at 4 hours post co-culture with 36% NL4-3 Jwt-ps20^inter ^donor cells at T:D ratio of 1:1 and 1:0.2. Data represent mean of three replicate assays. (C) Mean percentage of Gag^+ ^J-ps20^inter ^vs. J-ps20^high ^target cells at 24 hours post co-culture with 36% NL4-3 infected Jwt-ps20^inter ^donor cells at T:D ratio of 1:1 and 1:0.2. Data represent mean of three replicate assays. (D) Mean percentage of Gag^+ ^J-ps20^inter ^vs. J-ps20^high ^target cells at 4 hours post co-culture with YU2 infected Jwt-ps20^inter ^donor cells at T:D ratio of 1:0.2. Data represent mean of three replicate assays. (E) Target cells co-cultured with 40% YU2 infected donor cells were sorted for dye-positive single cells based on both FSC height vs. width followed by SSC height vs. width, on a BD FACS Aria II cell sorter. DNA extracted from these sorted singlet cells was subject to qDNA PCR for HIV-1 LTR. The level of HIV-1 LTR in J-ps20^inter ^vs. J-ps20^high ^target cells is shown relative to β-actin expression and normalized against DNA isolated from 8E5 cells. Asterisks denotes statistically significant data as calculated using an unpaired t-test (*P ≤0.05; **P ≤0.01; ***P ≤0.001)

We next probed the role of ps20 in cell-to-cell HIV transfer using a flow cytometry assay [10,12,15] (see Figure 1A). HIV-infected WT Jurkat cells (Jwt-ps20^inter^) served as infected donor cells. J-ps20^high ^and empty vector transduced J-ps20^inter ^target cells were co-cultured with donor cells that were 40% Gag^+ ^following infection with NL4-3 virus at 1:1 or 1:0.2 target:donor (T:D) cell ratios and the percentage of Gag^+ ^target cells enumerated at 4 (Figure 1B) and 24 hours (Figure 1C) post co-culture. At both time points and ratios tested, a higher proportion of Gag^+ ^cells were detected in J-ps20^high ^cells. However, a significant 2-fold difference between the J-ps20^high ^vs. J-ps20^inter ^population was only observed at the lower T:D ratio of 1:0.2, similar to our previous study that highlighted ps20-dependency of HIV-1 to be most marked at low virus challenge doses [23].

We next tested the ps20-dependency of an R5 HIV-1 strain (YU2) and additionally used a PCR-based assay to verify infection levels. Following co-culture with YU2 infected donor cells at a 1:0.2 T:D ratio, J-ps20^high ^targets had a 3-fold higher level of Gag transfer, after 4 hours compared to J-ps20^inter ^target cells (Figure 1C). In parallel, the co-cultured populations were FACS sorted for dye-positive single target cells and HIV-1 DNA measured in the sorted population. This sorting procedure ensured that infection levels were determined in single target cells, excluding possible target-target or donor-target conjugates [35], thereby providing an accurate estimation of infection in the infected target cells. qPCR on these samples showed a 6-fold higher level of HIV-1 LTR in the J-ps20^high ^vs. J-ps20^inter ^target cells (Figure 1D).

### HIV-1 transfer into J-ps20^high ^cells is fusion dependent and leads to productive infection

**Figure 2:**
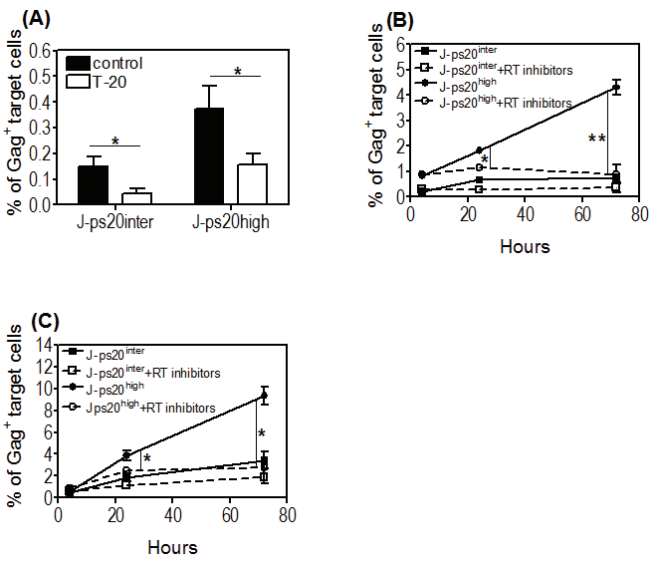
**HIV-1 transfer into J-ps20^high ^cells is dependent on virus fusion and leads to higher levels of productive infection**. (A) J-Ps20^high ^and J-ps20^inter ^target cells stained with DDAO SE vital dye were seeded at 1 × 10^5 ^cells per well of a 24 well plate in the presence or the absence of 5 μg/ml of T-20 for 1 hour prior to co-cultured with 18% Jwt ps20^inter ^NL4-3-infected donor cells at a T:D ratio 1:0.2. Mean percentage of Gag^+ ^J-ps20^inter ^vs. J-ps20^high ^target cells 4 hours post co-culture is shown. Data represent mean of three replicate assays. (B) The dye-labelled J-Ps20^high ^and J-ps20^inter ^target cells were seeded at 1 × 10^5 ^cells in the presence or the absence of 5 μM of RT-inhibitors (AZT+Lamimidine) for 1 hour prior to co-culture with 25% NL4-3-infected donor cells at a T:D ratio of (B) 1:0.2 or (C) 1:1. The percentage of Gag^+ ^J-ps20^inter ^vs. J-ps20^high ^target cells +/- RT inhibitors were assessed at 4, 24 and 72 hours post co-culture. Data represent the mean of three replicate assays. Asterisks denotes statistically significant data as calculated using a paired t-test (*P ≤0.05; **P ≤0.01).

Evidence exists for fusion -dependent and -independent T-T transfer of HIV-1 [6,36,37]. To probe this in the context of ps20, target cells were cultured with Jwt-ps20^inter ^donor cells productively infected with NL4-3 at a T:D ratio of 1:0:2 for 4 hours in the presence or absence of the T-20 fusion inhibitor. T-20 addition reduced virus transfer significantly by 3-fold and 2.4-fold in the J-ps20^inter ^vs. J-ps20^high ^cells, respectively (Figure 2A). To determine productive infection [38], target cells were cultured with reverse transcription RT inhibitors prior to co-culturing with Jwt-ps20^inter ^infected donor cells at a T:D ratio of 1:0.2 and Gag^+ ^cells enumerated at 4, 24, and 72 hours post co-culture. J-ps20^high ^target cells had higher infection with evidence of progressive increase in Gag^+ ^cells from the 4 to 72 hour time point, whereas there was no significant virus spread in the J-ps20^inter ^population (Figure 2B). The addition of RT inhibitors did not inhibit virus transfer in either population at 4 hours (Figure 2B). However, a significant reduction was observed in the J-ps20^high ^population with a 1.6-fold and 3-fold reduction between the J-ps20^high ^RT-inhibitor treated and untreated populations at 24 and 72 hours respectively (Figure 2B). RT-inhibitors have been noted not to influence HIV-1 transfer, but can inhibit Gag accumulation in prolonged co-cultures [37]. Our findings corroborate these observations. We next tested if increasing the virus challenge dose to 1:1 T:D ratio promoted virus spread in the J-ps20^inter ^cells. Figure 2C shows increase of Gag^+ ^cells at the 1:1 ratio from the 24-72 hour time point to be 1.83% (± 0.36) to 3.43% (± 0.78) respectively in J-ps20^inter ^cells, versus 3.84% (± 0.45) to 9.34% (± 0.79) respectively in J-ps20^high ^targets. At the lower T:D ratio, Gag^+ ^staining increased from 1.82% (± 0.13) to 4.3% (± 0.28) in J-ps20^high ^cells between 24-72 hours versus 0.66% (± 0.11) to 0.77% (± 0.05) in J-ps20^inter ^cells (Figure 2B). These data confirm J-ps20^inter ^cells require a higher virus challenge dose than J-ps20^high ^for efficient virus spread to be achieved in these cells.

### HIV-1 transfer correlates directly with ps20 expression in primary CD4^+ ^T cell clones

**Figure 3:**
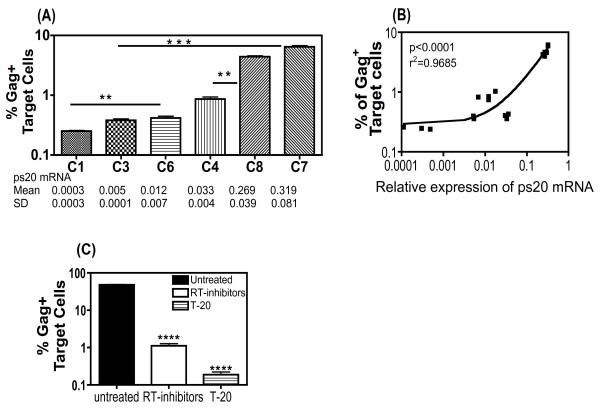
**HIV-1 transfer correlates directly with ps20 expression levels in primary CD4^+ ^T cell clones**. (A) Mean percentage of Gag^+ ^dye-labelled ps20^low^, ps20^inter ^and ps20^high ^primary target CD4^+ ^clones 4 hours post co-culture with 40% Jwt ps20^inter ^2044-infected donor cells at a T:D ratio of 1:0.2. Mean relative copy number of ps20 mRNA of each clone is given along the x-axis. (B) Correlation coefficient comparing the relative expression of ps20 in Clones 1,3,4,6,7,8 with their corresponding level of HIV-1 transfer 4 hours post co-culture with 40% Jwt ps20^inter ^2044-infected donor cells at a T:D ratio of 1:0.2. (C) Clone 7 (ps20^high^) was used as the target population and seeded at 1 × 10^5 ^cells in the presence or the absence of 5 μg/ml of T-20 or 5 μM of RT-inhibitors (AZT+Lamimidine) for 1 hour prior to co-culture with 40% 2044-infected donor cells at T:D ratio of 1:0.2. The percentage of Gag^+ ^target cells 48 hours post co-culture is shown. All data represent the mean of three replicate assays. Asterisks denotes statistically significant data as calculated using an unpaired t-test (Figure A), a two-tailed non-parametric Spearman's r correlation (Figure B) or paired t-test (Figure C). *P ≤0.05; **P ≤0.01; ***P ≤0.001; ****P ≤0.001.

A panel of six CD4^+ ^T cell clones from multiple donors (Additional file 1 figure S1D) were examined. Clones 1 (ps20^low^) and 6 (ps20^inter^) were gut-derived isogenic clones. Clones 3 (ps20^inter^), 7 (ps20^high^), 4 (ps20^inter^) and 8 (ps20^high^) were all blood-derived, with clones 3 and 7 being isogenic (Figure 3A). Cells were co-cultured for 4 hours with Jwt-ps20^inter ^donor cells that were 60% productively infected with the X4-HIV-1 strain, 2044 and in each case, ps20^high ^clones had a higher frequency of Gag^+ ^cells as compared to the ps20^inter ^or ps20^low ^counterparts. Differences between these clone pairs were as follows: 1.6-fold between clone 2 and clone 6, 16-fold between clone 3 and clone 7 and 5-fold between clone 4 and clone 8 (Figure 3A). Furthermore, comparison of all the ps20 low and intermediate clones (C1, C3, C4, C6) versus the ps20 high clones (C7, C8) highlighted statistically higher virus infection of the ps20 high clones (Mann-Whitney p = 0.0009) (Figure 3A). Indeed, a significant positive correlation was noted between HIV-1 transfer and ps20 mRNA expression in these clones (Two-tailed non-parametric Spearman's correlation, p < 0.0001, Figure 3B).

Virus transfer into primary clones was next confirmed to be fusion-dependent resulting in spreading infection. Representative Clone 7 (ps20^high^) was treated with either 5 μM RT-inhibitors or 5 μg/ml T-20 for 1 hour prior to co-culturing with 2044 infected Jwt-ps20^inter ^donor cells at a T:D ratio of 1:0.2 for 48 hours. The presence of RT inhibitors reduced Gag accumulation by 43-fold (Figure 3C). In the presence of the T-20 fusion inhibitor, inhibition was even more pronounced with a >100-fold reduction of Gag expression (Figure 3C). These data confirm that virus transfer into primary ps20^inter ^and ps20^high ^clones is fusion dependent and can lead to productive infection, with more marked suppression noted in ps20^high ^cells due to higher levels of virus transfer and spread in these cells.

### Blocking endogenous ps20 inhibits HIV-1 transfer in primary CD4^+ ^T cell clones

**Figure 4:**
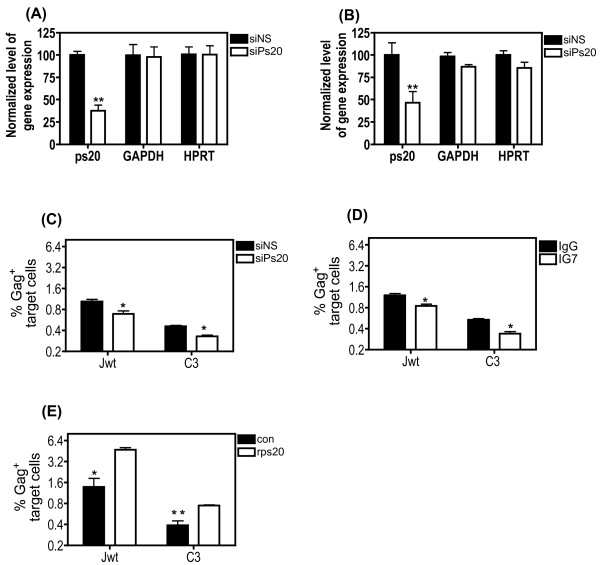
**Blocking endogenous ps20 inhibits HIV-1 transfer**. Jwt ps20^inter ^or clone 3 (ps20^inter^) was treated with either a non-silencing (siNS) siRNA or a WFDC1/ps20-silencing siRNA for 6 days. ps20, GAPDH and HPRT mRNA was then measured by qRT-PCR relative to β-actin expression in either (A) Jurkat population or (B) Clone 3. Normalized relative expression was calculated in reference to siNS control. (C) 8 × 10^4 ^siRNA treated cells were dye-labelled and co-cultured with 40% 2044-infected donor Jurkat cells at a T:D ratio of 1:0.2. The mean percentage of Gag+ target cells in a 4 hour transfer assay is shown. (D) 2 × 10^5 ^Jwt cells and C3 clone from were pre-cultured for 3 days with 5 μg/ml of either control mouse IgG1 or the anti-ps20 Ab IG7, then washed, dye-labelled and co-cultured with 40% 2044-infected donor cells at a T:D ratio of 1:0.2 in the presence of a further addition of each Ab. Mean percentage of Gag+ ps20high target cells is shown after 4 hours of co-culture. (E) 2 × 10^5 ^Jwt cells and C3 clone cells were cultured in the absence (control, con) or presence of 1 ug/ml of rps20 for 16 hours, washed, dye-labelled and co-cultured with donor cells infected with 40% 2044 at a T:D cell ratio of 1:0.2. The percentage of Gag+ ps20 target cells is shown after 4 hours of co-culture. All data represent the mean of three replicate assays. Asterisk denotes statistically significant data as calculated using a paired t-test. *P ≤0.05; **P ≤0.01.

Extensive characterisation of the Dharmacon Accell siRNA showed a consistent 50-60% specific knockdown of ps20 mRNA with maximal effects seen in ps20^inter ^populations. Accordingly, we conducted functional knockdown studies in the Jwt-ps20^inter ^and clone 3 (ps20^inter^). Both populations were treated with either non-specific (NS) siRNA or siRNA against ps20, which inhibited ps20 mRNA significantly by 62% in the Jurkat population and by 54% in clone 3 (Figure 4A, B respectively). To control for off target effects, GAPDH and HPRT expression was also measured relative to β-actin and no significant modulation of either noted in the presence of the siRNA against ps20 (Figure 4A, B). A reduction in ps20 expression was associated with a significant 34% and 28% reduction in virus transfer into the WT Jurkat cells and clone 3, respectively (Figure 4C). These observations were supported by antibody-mediated blocking experiments. A significant 29% and 36% reduction in virus transfer into Jurkat and clone C3 respectively was noted when cultured with anti-ps20 Ab relative to control IgG (Figure 4D). Conversely, recombinant ps20 (rps20) promoted virus transfer. Cells were pre-cultured with 1 ug/ml rps20 over-night, generated as previously described [23], washed to remove excess protein, then co-co-cultured with infected targets, resulting in a significant 3.4-fold and 1.9-fold enhancement of virus transfer into Jurkat and clone C3 respectively (Figure 4E). Similar observations of Ab-mediated blockade and rps20-induced transfer were also noted in additional clones (data not shown). These data confirm that blocking endogenous ps20 in primary CD4^+ ^T cells limits HIV-1 transfer.

### ps20^high ^CD4^+ ^T cells form a higher frequency of conjugates, multiple conjugates and virological synapse with HIV-1 infected donor cells

**Figure 5:**
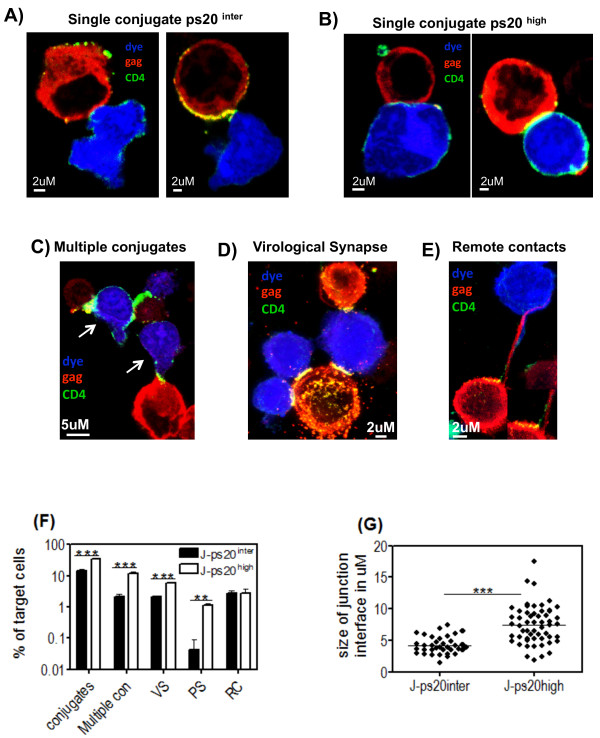
**ps20^high ^CD4+ T cells form a higher frequency of conjugates, multiple conjugates and VS with HIV-1 infected donor cells**. 1 × 10^5 ^DDAO vital dye labelled ps20^inter ^(Figure 5A) and ps20^high ^(Figure 5B) target cells were co-cultured with 60% 1 × 10^5 ^2044-infected donor cells for 1 hour on Poly-L-lysine coated glass cover slips. Cells were then fixed and stained with a PE anti-Gag (red) and a FITC anti-CD4 (green) Abs. The frequency of target cells that were involved in a single conjugate was defined as the percentage of dye-labelled target cells apposed to an HIV-1-infected donor cell. Representative high power fields captured at 63× magnification are shown, which depict single conjugates for between (A) J-ps20^inter ^or (B) J-ps20^high ^cells and HIV-infected donor cells. Left panels depict conjugates with no HIV-Gag CD4 polarization to conjugate interface. Right panels depict conjugates with HIV-Gag and CD4 polarization (yellow) to conjugate interfaces. (C) Picture shows a representative high power field of dye labelled targets (white arrows) involved in multiple conjugates, defined as a target cell apposed to two or more HIV-1-infected donor cells. (D) Picture shows a representative high power field of a polysynapse, defined as a cell with two or more virological synapses (yellow) at conjugate interfaces. (E) Picture shows a representative high power field of remote contacts (RC) (filopodial bridges or nanotubes) that connect uninfected target cells to HIV-infected targets. (F) Mean frequency of J-ps20^inter ^vs. J-ps20^high ^cells involved in single conjugate, multiple conjugates, or which contain virological synapses, polysynapses or are in contact through remote contacts are shown. A total of 600 random target cells were assessed across quadruplicate experiments. (G) Qualitative analysis of the junction diameter of a conjugates was measured using LEICA TCS SP2 software, where the diameter was measured across the medial section of a conjugate. Graph depicts the mean conjugate interface diameter (μM) between J-ps20^inter ^vs. J-ps20^high ^cells and HIV-1-infected donor cells. A total of at least 30 conjugates per population were measured across quadruplicate experiments. Asterisk denotes statistically significant data as calculated using an unpaired t-test (*P ≤0.05).

The quality and quantity of cell-cell conjugates formed in the presence of ps20 were next assessed. To avoid inherent differences in primary clonal populations, these studies were performed using the Jurkat model system. First the number of conjugates formed was assessed. Conjugates were defined as a target cell closely apposed to an infected donor cell, and multiple conjugates (MCs) as a target cell closely apposed to two or more infected donor cells (Figures 5A, B, C). We observed a significant 2.3-fold and 5.4-fold higher frequency of conjugate and MC formation and a 2.75-fold higher frequency of Gag and CD4 polarization to conjugate interfaces (VS formation - see Figure 5D) in J-ps20^high ^vs. J-ps20^inter ^populations respectively (Figure 5F). However, the proportion of conjugates containing VS was similar, 14.6% vs. 17.3% in J-ps20^inter ^vs. J-ps20^high ^conjugates, in keeping with the notion that the number of VS formed is determined by the number of conjugates. Previously, it has been shown postulated that the formation of multiple virological synapses (termed polysynapses-PS) in conjugates of uninfected targets and HIV-infected donors is an efficient mode of virus dissemination [12]. We, therefore, enumerated conjugates (target or donor) containing two or more synapses simultaneously (Figure 5D). A marked 28-fold higher frequency of PS in co-cultures of J-ps20^high ^vs. J-ps20^inter ^cells was noted (Figure 5F). However, the frequency of remote contacts (filopodial bridges and nanotubes) formed between uninfected target and infected donor cells did not differ between J-ps20^high ^vs. J-ps20^inter ^cells (Figure 5F). Interestingly, ps20^high ^cells were observed to be more closely apposed to infected donor cells compared to ps20^inter ^cells (Figure 5A vs. 5B). To quantify this observation, the medial diameter of conjugate interfaces was measured and found to be significantly larger in conjugates with ps20^high ^targets. J-ps20^high ^vs. J-ps20^inter ^conjugates had a mean diameter of 7.46 uM (± 0.41) vs. 4.25 uM (± 0.23) respectively (Figure 5G). Together, these data highlight ps20 to impact the fundamental biologic process of cell-cell conjugation.

### ps20 promotes conjugate and multiple conjugate formation more effectively than ICAM-1

**Figure 6:**
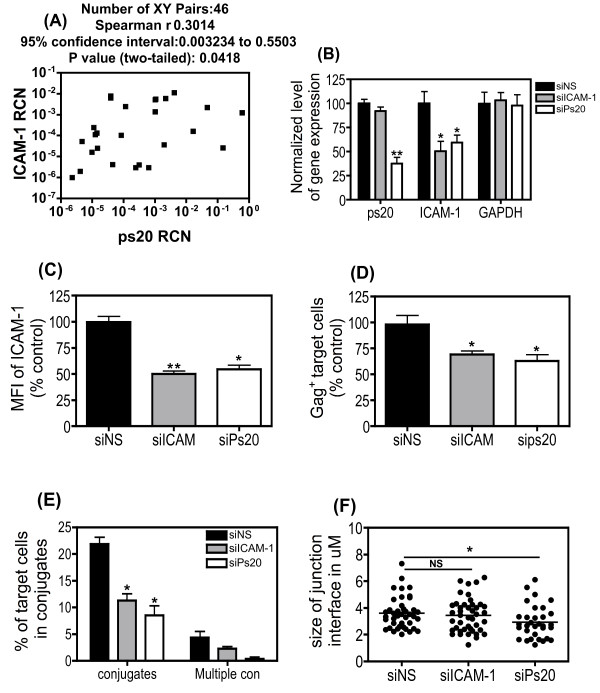
**siRNA-mediated knockdown of ps20 inhibits conjugate and multiple conjugate formation more effectively than siRNA-mediated knockdown of ICAM-1**. (A) ps20 and ICAM-1 mRNA levels were measured in a selection of ps20^high ^and ^ps20inter/low ^cells at 4 different time points. Data show a two-tailed non-parametric Spearman's r correlation of all data points. CD4 T-cell Jwt ps20^inter ^were treated with either; a non-silencing (NS), ICAM-1 or WFDC1/ps20-silencing siRNA pool for 6 days. (B) After siRNA treatment the expression of ICAM-1, ps20 and GAPDH mRNA was analyzed by qRT-PCR and relative expression to β-actin was measured. Normalized relative expression was calculated in reference to siNS control. Data represent the mean of three replicate assays. (C) Surface expression of ICAM-1 in siRNA treated cells is shown as assessed by standard immunofluorescence. Normalized MFI was calculated in reference to siNS control. Data represent the mean of three replicate assays (D) 8 × 10^4 ^NS, ICAM-1, or ps20 siRNA-treated WT Jurkat cells were dye-labelled and co-cultured with donor 40% 2044-infected donor cells at a T:D ratio of 1:0.2. Mean percentage of Gag^+ ^target cells after 4-hour co-culture is shown. Normalized % of Gag^+ ^target cells was calculated in reference to siNS control. Data represent the mean of three replicate assays. (E) 5 × 10^5 ^siRNA treated cells were dye-labelled and co-cultured with 5 × 10^5 ^60% 2044-infected donor cells. Co-cultures were incubated for 1 hour on Poly-L-lysine coated glass cover slips, then fixed and stained with a FITC anti-Gag (Green) Ab. Conjugates and MCs were assessed as before in at least 500 random target cells per population across triplicate experiments. (F) The panel depicts the mean conjugate interface diameter (μM) between siRNA treated Jurkat cells and HIV-1-infected donor cells. A total of at least 30 conjugates per population were measured across triplicate experiments. Asterisk denotes statistically significant data as calculated using a paired t-test (*P ≤0.05) in relation to NS siRNA control.

We assessed the potency of ps20- vs. ICAM-1- mediated virus transfer and determined the relative importance of each in T-T conjugate formation, using an si-RNA targeted knock-down strategy in the Jurkat system. Treatment of Jwt-ps20^inter ^cells with siRNA against ICAM-1 led to a significant 50% reduction in the levels of ICAM-1 mRNA, with no significant reduction of either ps20 or GAPDH expression (Figure 6A). However, siRNA against ps20 led to a significant 60% reduction in ps20 mRNA, and a concomitant 40% reduction in ICAM-1 mRNA, with no reduction in GAPDH. This confirms our previous observations that blocking ps20 can inhibit ICAM-1 expression [23]. Surface ICAM-1 protein expression was reduced by 50% and 45% with siRNA against ICAM-1 or ps20, respectively (Figure 6B). Ps20 vs. ICAM-1 knockdown resulted in a 36% vs. 30% reduction in the levels of virus transfer respectively (Figure 6C). In addition, ICAM-1 versus ps20 siRNA inhibited single conjugates by 50% vs. 61% respectively (Figure 6D). ICAM-1 siRNA inhibited multiple conjugates by 50% versus a marked 92% by ps20 siRNA (Figure 6D). Lastly, the size of the conjugate interface was not affected by ICAM-1 knockdown, whereas ps20 knockdown had a small but consistent effect; a significant 1.2-fold reduction in mean conjugate diameter from 3.601 (± 0.1871) μm in NS siRNA treated control to 2.933 (± 0.2179) μm in ps20 siRNA treated cells was noted (Figure 6E).

## Discussion

Cell-to-cell HIV transfer is a significant mode of virus spread amongst CD4^+ ^T cells *in-vitro *[3-10] and also likely to be predominant *in vivo*, since memory CD4^+ ^T cells are more likely to become infected while trafficking through secondary lymphoid tissue, where lymphocyte velocities decrease allowing for cell-virus and cell-cell interactions to take place [10,39,40]. Therefore, identifying host factors that regulate this process is of importance to understanding HIV-1 dissemination *in-vivo*.

This paper highlights ps20 to be a novel rate-limiting step in T-T HIV-1 transfer. We demonstrate this by utilising a flow-cytometry and a PCR-based HIV transfer assay in a ps20 transduced Jurkat model system, as well as in a panel of primary CD4^+ ^T cell clones. We report a significant positive correlation between endogenous ps20 expression levels and T-T virus transfer. Blocking ps20 activity with siRNA or specific antibody significantly inhibited T-T transfer. Conversely, gain of function studies using ps20-transduced Jurkat CD4^+ ^T cells or the exogenous addition of rps20 confirmed ps20 to promote HIV-1 transfer. We further show inhibition of virus transfer and spread into ps20^high ^cells by the T-20 fusion inhibitor and RT inhibitors respectively, with differences in virus spread between ps20^high ^vs. ps20^low/inter ^populations reaching upto 5.7-fold in Jurkat cells (Figure 2) and 8.7-fold in primary clones (Figure 3), highlighting ps20 to be a potentially potent pathway in promoting T-T virus dissemination.

Divergent data exist with regard to cell-cell transfer mediated by fusion vs. endocytosis contributing to productive HIV infection [6-8,36,37,41]. In studies using unstimulated CD4^+ ^T-cells as target populations, virus transfer through endocytosis [8,36,37] was noted. However, other studies show T-T virus transfer to be both co-receptor- and virus fusion- dependent [6,41]. Whilst the infected donor cell in these divergent studies was Jurkat or Molt 4, a key difference appears to be the state of target cell activation with evidence of fusion dependent entry into activated memory CD4^+ ^T-cells targets [41]. Our observations with activated clonal CD4 T-cells or Jurkat cells are therefore compatible with this data. Taken together, these findings suggest that the activation state of the target cell may account for observed differences in the mode of virus uptake during cell-cell virus transfer. Indeed, these differences may account for other data showing ICAM-1 and LFA-1 as not being critical for HIV transfer to unstimulated *ex vivo *CD4 T cells [38], which have been shown to take up virus via endocytosis [8,36,37]. Thus it is reasonable to hypothesize that, differences in the molecular determinants and the mechanisms that govern virus transfer are at least partly dependent on the state of activation of the target CD4^+ ^T-cell.

An important step in HIV transfer is the conjugation between an infected and an uninfected cell, leading to VS formation, through which virus can be directly transferred [3-10]. A time-lapse microscopy study highlighted that up to 80% of T-T conjugates, at some point after conjugation/contact, form a VS [11]. Consequently, we examined the role of ps20 in the quality and quantity of T-T conjugate formation. Evidence is provided in support of ps20 promoting intercellular conjugation and VS formation. As the overall proportion of conjugates containing VS was similar between ps20^high ^and ps20^low ^populations, the capacity of ps20 to promote VS formation may be attributable to the protein enhancing T-cell conjugation. In addition, we observed a higher frequency of multiple conjugates and polysynapses in the presence of ps20. Therefore, the ability of ps20 to promote multiple conjugates and polysynapses may be of critical importance to virus dissemination in lymphoid and mucosal tissue by allowing for fewer transient interactions between cells [see [12]]. This notion is further supported by the observation that the conjugate interface between infected donor cells and ps20^high ^targets was significantly larger compared to ps20^low ^targets. These characteristics were attributable to ps20, since knocking down ps20 expression significantly reduced the number of single conjugates, multiple conjugates, the size of the conjugate interface and resulting virus transfer.

Molecular determinants of cell-cell conjugate and VS formation include the actin and microtubule cytoskeletal networks, cell signalling, tetraspanin and lipid raft recruitment [6,13-22,42]. In addition to the HIV-1 receptor complex, several adhesion molecules can polarize to, and stabilize these supramolecular synapses [13,15,17,20,21]. In particular, Jolly et al. showed that anti-ICAM-1 inhibited VS by 30%, while anti-LFA-1 inhibited VS and conjugate formation from 15-90% depending on the blocking Ab used [17]. Previously, we demonstrated that ps20 enhanced HIV-1 infection through ICAM-1 modulation [23]. Here we confirm and extend these observations. Knocking down ps20 by 60% specifically suppressed ICAM-1 mRNA by 40% and ICAM-1 surface staining by 45% and both ICAM-1 and ps20 individually contributed to conjugate formation and virus transfer. Strikingly, knocking down ps20 inhibited multiple conjugate formation by 90% compared to 50% inhibition by siRNA against ICAM-1. Furthermore, ICAM-1 did not impact the size of the conjugate interface, whilst ps20 did so, albeit marginally (Figure 6E). These data suggest that ps20-induced ICAM-1 modulation though important, may not fully account for the ps20 effect. As ps20 is implicated in regulating extracellular matrix (ECM) components [31,34] and given recent data that another retrovirus, HTLV-1, can be captured, and then transmitted through ECM glycoproteins' [43], the identification of other adhesion and ECM targets regulated by ps20 and their role in cell-cell HIV-1 transfer could enhance understanding of mechanisms that drive HIV-1 dissemination *in-vivo*.

The molecular mechanisms by which ps20 regulates ICAM-1 expression and clustering through putative binding partners and signalling functions are part of on-going work in our laboratory. Other work highlights a fundamental role for ps20 in cell migration and angiogenesis [31]. Both these processes are recognised to modulate adhesion molecules [32,33]. As cell-cell adhesion plays a significant role in successful virus infections in general [44], it could be argued that the potency of ps20 to promote HIV-1 infection is linked with it's fundamental role in regulating cell adhesion. The novel observation that CD4^+ ^T cells can be segregated into stable subsets on the basis of ps20 expression coupled with the observation that ps20^high ^CD4^+ ^T cells are more susceptible to infection than ps20^low ^cells, strongly suggests that ps20^+ ^CD4^+ ^T cells may be preferentially targeted and lost *in-vivo*. Our contention is that local concentrations of ps20, in tissue such as the gut, may drive HIV-1 infection and CD4^+ ^T cell loss by increasing adhesion antigen expression on CD4^+ ^T cells through autocrine and paracrine effects, thereby highlighting a novel role for the ancient whey acidic protein, WFDC1/ps20, in HIV-1 pathogenesis.

## Conclusions

This study highlights three novel aspects of T-T HIV transfer. First, using three approaches to probe T-T HIV transfer, namely, flow cytometry, PCR and confocal microscopy, this study highlights ps20 to be a novel host factor that promotes cell-cell conjugation and virological synapse formation in gain and loss of function assay systems. Second, one mechanism by which ps20 promotes intercellular HIV transfer is by regulating surface ICAM-1 expression levels. Importantly, ps20 promoted multiple cell conjugation more efficiently than ICAM-1 and was identified to promote poly-synapse formation. Host factors that promote poly-synapse formation may be particularly potent in promoting virus dissemination *in vivo *[12] and thereby impact HIV-1 pathogenesis. Thirdly, the observation that primary CD4 T-lymphocyte clones segregate naturally into distinct subsets based on endogenous ps20 expression and that ps20 levels correlate with intercellular HIV transfer, identifies ps20 to a novel marker of CD4 T cells that are vulnerable to HIV infection. Together, these observations highlight that ps20 is a novel host factor that could promote virus dissemination by promoting T-T cell conjugation.

## Methods

### CD4^+ ^T cells

Jurkat CCR5^+ ^T cells (from National Centre for Biological Standards & Controls, NIBSC, UK) were maintained in RPMI 1640 (GIBCO Invitrogen, UK) + 10% Fetal Calf Serum (FCS) (Helena Biosciences UK), 20 ug/ml Gentamycin (Sigma-Aldrich, UK). A panel of random blood CD4+ T-cell clones generated by standard limiting dilution cloning [23,45] was screened for ps20 mRNA, and cells were identified to be ps20^high^, ps20^inter^, ps20^low ^(see Additional file figure S1A). Additionally, two CD4^+ ^T cell clones isolated from Endoscopy sections taken from non-lesional or lesional portion of the colon of a patient with Crohns disease (kind gift Dr. Deena Gibbons and Prof. Adrian Hayday King's College London) were identified to be ps20^low ^and ps20^high ^respectively. Clones were maintained in RPMI 1640, 10% FCS + 10% Human AB^+ ^Serum (First Link, UK), 20 ug/ml Gentamycin. A typical feeding cycle of CD4^+ ^T cell clones included activation with irradiated allogeneic PBMC (1:1 ratio) + 2 μg/ml PHA (Biostat Diagnostic Systems, Germany) + 20 IU/ml IL2 (Proleukin, Chiron, UK) every 10-14 days. Cells were split and fed every 3-4 days with fresh 30 IU/ml IL2.

### Stable ps20^high ^Jurkat CD4^+ ^T cells

The *WFDC1 *gene was digested out from the pHA/*WFDC1 *expression plasmid (kind gift D. Rowley, Baylor University, USA) with *Eco*RI and cloned into an MMLV based bi-cistronic retroviral vector, pCxCR encoding red fluorescent protein (RFP) under the control of a cytomegalovirus (CMV) promoter (kind gift Greg Towers, University College London). Retroviral particles encoding WFDC1/RFP (pCps20CR), or RFP alone (pCxCR, empty vector control) were made by transient transfection of 293T cells with pCxCR, or pCG9CR, along with the packaging construct pCpg (MMLV Gag/Pol) and an envelope construct encoding VSV-G (pMD.G) (kind gift D. Trono, Geneva, Switzerland). Retroviral particles were harvested 48 hrs after transfection, clarified and titrated onto 2 × 10^5 ^CCR5^+ ^CD4^+ ^Jurkat cells, three times over 3 days. Cells were sorted for RFP, expanded and ps20 expression confirmed by qRT-PCR. The ps20 transduced population is referred to as J-ps20^high^; the empty vector control as J- ps20^inter^.

### Virus Production

The primary HIV-1 X4 strain 2044 was propagated in PHA activated PBMC [35]. The HIV-1 full-length molecular clones NL4-3 and YU2 (kind gift, M Malim, King's College London) were produced by the transient transfection of 293T cells, using Fugene 6 reagent (Roche, Switzerland). Viral stocks were standardised based on Gag p24 concentrations determined by p24 ELISA (NIH Reagents).

### Antibodies

Anti-CD54 RPE (clone 15.2; Serotec, U.K.); anti-ps20 (IG7; kind gift D. Rowley); anti-ICAM-1 Ab (LB-2) (BD Pharmingen, CA); FITC- or PE- conjugated Gag-p24 antibody (clone KC57; Beckman Coulter, UK) were used. In addition: anti-CD4 (L120), anti-HIV ENV and rabbit anti-HIV Gag p24 and p17 were obtained from NIBSC, UK. Isotype-matched mAbs were from BD Pharmingen, CA.

### Ps20 ELISA

Affinity purified polyclonal rabbit anti-ps20 antibody (202-254) specific for residues 206-220 of the ps20 amino acid sequence was generated through Eurogentec Ltd, Belgium. Nunc 96-well plates were coated over-night at 4°C with 1 ug/mL 202-254 diluted in PBS. Plates were blocked with PBS, 1% BSA for 2H at room temperature (RT), washed x3 with PBS-0.2% Tween-20 and test samples added for 2 hours at RT. Plates were washed 6 times to remove unbound material. Detection was with 2 ug/ml IG7 anti-ps20 conjugated to horseradish peroxidase in PBS-1% BSA-0.2% Tween-20 for 2 hours, RT followed by further 6 washes. 150 μL of substrate OPD (Sigma) was added for 30 min. at room temperature and stopped with 50 μL of 4 M sulphuric acid. Optical densities were determined at 492 nm in a Bio-Rad ELISA plate reader. Ps20 concentrations were determined in relation to a standard curve using recombinant ps20 of known concentration generated in drosophila^2^.

### Spreading HIV-1 infection

Cells were challenged with virus stocks standardised on Gag p24 concentration, and unbound virus was removed by washing after 24 hours. Productive infection was monitored by intracellular staining for HIV-1 Gag p24 using a Fix and Perm kit (AD Serotec, U.K.). Cells were fixed for 10 minutes at RT in fixation buffer, washed once with cold PBS, then resuspended in permeabilization buffer and a 1/10 final dilution of KC57 RD1 or FITC added for 25 minutes at RT. Samples were washed twice with PBS and resuspended in PBS 2%FCS, 2% paraformaldehyde and analysed on a FACSCalibur instrument and data analysed using Flow Jo software.

### Cell to cell HIV-1 transfer assay

A modified version of an assay described by Sourisseau *et al *[10,15] was used. Briefly, WT Jurkat CD4^+ ^T cells (Donor) were infected with HIV-1 strains till cultures were 10-60% Gag p24+. Targets cells comprising ps20^high^, ps20^inter ^or ps20^low ^CD4^+ ^T cell population were first labelled with the Cell Trace FarRed DDAO-SE vital dye (Invitrogen UK). 1 × 10^6^/ml target cells were incubated in PBS with 10 μM of the DDAO-SE dye for 7 minutes at 37°C, washed twice in PBS 5% FCS and co-cultured with infected donor cells at varying ratios in a final volume of 500 μl in a 24-well plate. Infection inhibitors included were 5 μg/ml T-20 (Roche, Hertfordshire, UK), or 5 μM each of AZT/Lamirudine (AIDS Repository Reagent Program, MD, USA). Gag transfer was measured 4 hours post co-culture by enumerating dye-labelled targets cells that stained positive for HIV-1 Gag p24 by flow cytometry.

### siRNA knockdown

Accell siRNA smart pools targeting ps20 (E-013097-00), ICAM-1 (E-003502-00-0010) or a non-specific targeting control (D-001910-10) were purchased from Dharmacon, CO, USA. Cell populations were washed 3 times in PBS prior to resuspension at a density of 3 × 10^5^/ml in Accell siRNA passive uptake media containing, 3% FCS and 1 μM of specified Accell siRNA pools. For primary clones, the medium was supplemented with 30 IU/ml IL-2. 3 days later cells were washed and re-incubated at 3 × 10^4 ^cells/well in a 96-well plate in a fresh aliquot of complete passive uptake media containing 1 μM of specified Accell siRNA pool. 3 days later, cells were washed, and target gene knockdown efficiency was assessed by qRT-PCR before use in functional assays.

### mRNA measurement

Total RNA was extracted using an RNaeasy kit (Qiagen, UK), then converted to cDNA (Ambion, Inc., TX, USA). ps20 was measured using a custom designed Taqman™ assay as described previously [23]. ICAM-1 was measured using a TaqMan^® ^primer and probe set (Hs99999152_m1). ps20/ICAM-1 expression was measured relative to HPRT 1(Hs01003268_g1) or β-actin (Hs99999903_m1) or GAPDH (Hs99999905_m1) (TaqMan^®^; Applied Biosystems, UK), according to manufacturer's instructions, on an ABI Prism 7900 HT (Applied Biosystems, CA, USA). Data was analyzed using SDS 2.3 software (Applied biosystems, CA, USA). Relative copy number (RCN) was calculated by determining the delta ct values, which was used in the following equation (RCN) = 2^ ^(-ΔCt)^.

### HIV-1 LTR measurement

DNA was extracted using a DNeasy kit (Qiagen, UK). Primer sequences for HIV-1 LTR were; L2:CTGTGGATCTACCACACACAAGGCTAC (forward) and L3:GCTGCTTATATGTAGCATCTGAGGGC (reverse) [46] and measured using TaqMan^® ^probe assay relative to β-actin (Applied Biosystems, CA, USA). Delta Ct was first calculated, then normalized by subtracting the delta ct values generated from 8E5 cells, which contain one integrated copy of HIV-1. Relative copy number = 2^ ^(-ΔΔCt)^.

### Confocal microscopy

Far Red DDAO-SE dye-labelled target cells were cultured with HIV-1 infected donor cells along with 5 ug/ml anti-CD4 and anti-HIV ENV Abs on poly-L-lysine coated glass cover slips (Sigma, UK). Cells were washed twice with PBS, fixed for 10 minutes in 4% paraformaldehyde, 1% BSA in PBS at RT, washed twice with PBS 1% BSA and permeabilized using 0.2% triton X-100 for 10 minutes at RT. The cells were then blocked and quenched in 50 mM NH_4_CL, 2% mouse serum, 2% BSA, 0.05% sodium azide. Cells were then stained with an anti-Gag Ab, KC57-PE (Beckman Coulter), and an anti-CD4, L120-FITC (BD, San Jose, CA) at 1/20 total volume dilutions in block/quench buffer for 60 minutes at RT. After incubation, cells were washed 3× with PBS and mounted on Superfrost Plus glass slides (VWR International, UK), using ultramount aqueous permanent mounting medium (Dako, Denmark). Slides were allowed to dry overnight at 4°C in the dark. The next day the cells were visualized by scanning laser confocal microscopy on a Leica DM IR2 (LEICA Microsystems, Germany) and analyzed using LEICA TCS SP2 confocal software (LEICA, Microsystems, Germany). Images were acquired using a 63× oil-immersion objective and processed using Adobe Photoshop CS (Adobe Systems, San Jose, CA).

### Statistical Analysis

Statistical analysis of data was performed using Graph Pad PRISM Software, San Diego, CA. All p-values reported are two-tailed. P values of less than or equal to 0.05 were deemed significant.

## Conflict of interests

The authors declare that they have no competing interests.

## Authors' contributions

AV was involved in experimental design, hypothesis generation and manuscript editing. RAA conducted 80% of the work outlined and wrote the manuscript. JLR helped with qRT-PCR. GT helped with qDNA-PCR and HIV transfer assays. SKR helped develop the ps20 ELISA. All authors read and approved the final manuscript.

## Supplementary Material

Additional file 1 **Figure S1. Jurkat CD4 T cells stably transduced to express full-length human ps20 are more susceptible to cell-free HIV-1 infection**. A) The mean relative copy number (RCN) of ps20 mRNA (relative to the HPRT house keeping gene) measured in three biological replicate samples by qRT-PCR; in the empty vector (EV) transduced ps20^inter ^Jurkat cells, WFDC1/ps20 transduced ps20^high ^Jurkat cells, wild-type Jurkat cells as well as a panel of 10 primary CD4^+ ^T-cell clones (C1-C10) is shown. The two horizontal lines are arbitrarily set to define CD4 T-lymphocyte populations as ps20 low (RCN <0.001), ps20 intermediate (RCN 0.001-0.1) and ps20 high (>0.1RCN). (B) Mean secreted ps20 protein levels measured in three replicate samples by a ps20 specific ELISA assay are shown. Positive control was supernatant from the human embryonic kidney cell line, 293T cells transfected with a WFDC1/ps20 encoding construct (pBKps20) and supernatant from ps20 mRNA high HeLa cells; negative control was supernatant from 293T cells transfected with empty vector alone (pBK). Test samples included 48-hour culture supernatant from the WFDC1/ps20 transduced population and the empty vector transduced EV population. (C) 2 × 10^5 ^J-ps20^inter ^or J-ps20^high ^cells were challenged with 2.5 ng/1 × 10^6 ^cells of the X4-tropic HIV-1 strain, NL4-3. Productive infection was measured by intracellular staining for HIV-1 p24 capsid antigen and the percentage of p24 positive cells determined on days 3, 5 and 7 post-challenge. (D) J-ps20^inter ^or J-ps20^high ^cells were first pre-cultured overnight (16 hrs) in 5 μg/ml of either control mouse IgG_1 _or the anti-ps20 Ab, IG7, then challenged with NL4-3 for 24 hours (10 ug Gag p24 antigen concentration of virus stock/10^5 ^cells). 24 hours later equivalent numbers of cells were trypsinized to remove surface bound virus, washed and cell pellets lysed in PBS with 10% triton-X 100. The amount of Gag p24 antigen was then measured by ELISA and used to assess the fold increase in infection over background. The mean and standard error of triplicate replicate experiments are shown. Click here for file

## References

[B1] Sherer NM Lehmann MJ Jimenez-Soto LF Horensavitz C Pypaert M Mothes W Retroviruses can establish filopodial bridges for efficient cell-to-cell transmission Nat Cell Biol 2007 9 310 315 10.1038/ncb1544 17293854 PMC2628976

[B2] Sowinski S Jolly C Berninghausen O Purbhoo MA Chauveau A Köhler K Oddos S Eissmann P Brodsky FM Hopkins C Onfelt B Sattentau Q Davis DM Membrane nanotubes physically connect T cells over long distances presenting a novel route for HIV-1 transmission Nat Cell Biol 2008 10 211 219 10.1038/ncb1682 18193035

[B3] Jolly C Sattentau QJ Retroviral spread by induction of virological synapses Traffic 2004 5 643 650 10.1111/j.1600-0854.2004.00209.x 15296489

[B4] Sattentau Q Avoiding the void: cell-to-cell spread of human viruses Nat Rev Microbiol 2008 6 815 826 10.1038/nrmicro1972 18923409

[B5] Dimitrov DS Willey RL Sato H Chang LJ Blumenthal R Martin MA Quantitation of human immunodeficiency virus type 1infection kinetics J Virol 1993 67 2182 2190 8445728 10.1128/jvi.67.4.2182-2190.1993 PMC240333

[B6] Jolly C Kashefi K Hollinshead M Sattentau QJ HIV-1 cell-to-cell transfer across an Env-induced, actin-dependent synapse J Exp Med 2004 199 283 293 10.1084/jem.20030648 14734528 PMC2211771

[B7] Carr JM Hocking H Li P Burrell CJ Rapid and efficient cell-to-cell transmission of human immunodeficiency virus infection from monocyte-derived macrophages to peripheral blood lymphocytes Virology 1999 265 319 329 10.1006/viro.1999.0047 10600603

[B8] Chen P Hubner W Spinelli MA Chen BK Predominant mode of human immunodeficiency virus transfer between T cells is mediated by sustained Env-dependent neutralization-resistant virological synapses J Virol 2007 81 12582 12595 10.1128/JVI.00381-07 17728240 PMC2169007

[B9] Sato H Orenstein J Dimitrov D Martin M Cell-to-cell spread of HIV-1 occurs within minutes and may not involve the participation of virus particles Virology 1992 186 712 724 10.1016/0042-6822(92)90038-Q 1370739

[B10] Sourisseau M Sol-Foulon N Porrot F Blanchet F Schwartz O Inefficient human immunodeficiency virus replication in mobile lymphocytes J Virol 2007 81 1000 1012 10.1128/JVI.01629-06 17079292 PMC1797449

[B11] Hübner W McNerney GP Chen P Dale BM Gordon RE Chuang FY Li XD Asmuth DM Huser T Chen BK Quantitative 3D video microscopy of HIV transfer across T cell virological synapses Science 2009 323 1743 1747 10.1126/science.1167525 19325119 PMC2756521

[B12] Rudnicka D Feldmann J Porrot F Wietgrefe S Guadagnini S Prévost MC Estaquier J Haase AT Sol-Foulon N Schwartz O Simultaneous HIV Cell-to-Cell Transmission To Multiple Targets Through Polysynapses J Virol 2009 83 6234 46 10.1128/JVI.00282-09 19369333 PMC2687379

[B13] Nobile C Petit C Moris A Skrabal K Abastado JP Mammano F Schwartz O Covert human immunodeficiency virus replication in dendritic cells and in DC-SIGN-expressing cells promotes long-term transmission to lymphocytes J Virol 2005 79 5386 5399 10.1128/JVI.79.9.5386-5399.2005 15827153 PMC1082762

[B14] Vasiliver-Shamis G Cho MW Hioe CE Dustin ML Human immunodeficiency virus type 1 envelope gp120-induced partial T-cell receptor signalling creates an F-actin-depleted zone in the virological synapse J Virol 2009 83 11341 11355 10.1128/JVI.01440-09 19710135 PMC2772796

[B15] Sol-Foulon N Sourisseau M Porrot F Thoulouze MI Trouillet C Nobile C Blanchet F di Bartolo V Noraz N Taylor N Alcover A Hivroz C Schwartz O ZAP-70 kinase regulates HIV cell-to-cell spread and virological synapse formation EMBO J 2007 26 516 526 10.1038/sj.emboj.7601509 17215865 PMC1783460

[B16] Jolly C Sattentau QJ Human immunodeficiency virus type 1 assembly, budding, and cell-cell spread in T cells take place in tetraspanin-enriched plasma membrane domains J Virol 2007 81 7873 7884 10.1128/JVI.01845-06 17522207 PMC1951303

[B17] Jolly C Mitar I Sattentau QJ Adhesion molecule interactions facilitate human immunodeficiency virus type 1-induced virological synapse formation between T cells J Virol 2007 81 13916 13921 10.1128/JVI.01585-07 17913807 PMC2168851

[B18] Arthos J Cicala C Martinelli E Macleod K Van Ryk D Wei D Xiao Z Veenstra TD Conrad TP Lempicki RA McLaughlin S Pascuccio M Gopaul R McNally J Cruz CC Censoplano N Chung E Reitano KN Kottilil S Goode DJ Fauci A SHIV-1 envelope protein binds to and signals through integrin alpha4beta7, the gut mucosal homing receptor for peripheral T cells Nat Immunol 2008 9 301 309 10.1038/ni1566 18264102

[B19] Krementsov DN Weng J Lambele M Roy NH Thali M Tetraspanins regulate cell-to-cell transmission of HIV-1 Retrovirology 2009 6 64 10.1186/1742-4690-6-64 19602278 PMC2714829

[B20] Tsunetsugu-Yokota Y Yasuda S Sugimoto A Yagi T Azuma M Yagita H Akagawa K Takemori T Efficient virus transmission from dendritic cells to CD4+ T cells in response to antigen depends on close contact through adhesion molecules Virology 1997 239 259 268 10.1006/viro.1997.8895 9434717

[B21] McDonald D Wu L Bohks SM KewalRamani VN Unutmaz D Hope TJ Recruitment of HIV and its receptors to dendritic cell-T cell junctions Science 2003 300 1295 1297 10.1126/science.1084238 12730499

[B22] Jolly C Sattentau QJ Human immunodeficiency virus type 1 virological synapse formation in T cells requires lipid raft integrity J Virol 2005 79 12088 12094 10.1128/JVI.79.18.12088-12094.2005 16140785 PMC1212596

[B23] Alvarez R Reading J King DF Hayes M Easterbrook P Farzaneh F Ressler S Yang F Rowley D Vyakarnam A WFDC1/ps20 is a novel innate immunomodulatory signature protein of human immunodeficiency virus (HIV)-permissive CD4+ CD45RO+ memory T cells that promotes infection by upregulating CD54 integrin expression and is elevated in HIV type 1 infection J Virol 2008 82 471 486 10.1128/JVI.00939-07 17942534 PMC2224370

[B24] Tardif MR Tremblay MJ LFA-1 is a key determinant for preferential infection of memory CD4+ T cells by human immunodeficiency virus type 1 J Virol 2005 79 13714 13724 10.1128/JVI.79.21.13714-13724.2005 16227291 PMC1262559

[B25] Liao Z Roos JW Hildreth JE Increased infectivity of HIV type 1 particles bound to cell surface and solid-phase ICAM-1 and VCAM-1 through acquired adhesion molecules LFA-1 and VLA-4 AIDS Res Hum Retroviruses 2000 16 355 366 10.1089/088922200309232 10716373

[B26] Fortin JF Cantin R Lamontagne G Tremblay M Host-derived ICAM-1 glycoproteins incorporated on human immunodeficiency virus type 1 are biologically active and enhance viral infectivity J Virol 1997 71 3588 3596 9094631 10.1128/jvi.71.5.3588-3596.1997 PMC191506

[B27] Tardif MR Tremblay MJ Presence of host ICAM-1 in human immunodeficiency virus type 1 virions increases productive infection of CD4+ T lymphocytes by favoring cytosolic delivery of viral material J Virol 2003 77 12299 12309 10.1128/JVI.77.22.12299-12309.2003 14581566 PMC254246

[B28] Bingle CD Vyakarnam A Novel innate immune functions of the whey acidic protein family Trends Immunol 2008 29 444 453 10.1016/j.it.2008.07.001 18676177

[B29] Ranganathan S Simpson KJ Shaw DC Nicholas KR The whey acidic protein family: a new signature motif and three-dimensional structure by comparative modeling J Mol Graph Model 1999 17 106 10.1016/S1093-3263(99)00023-6 10680116

[B30] Moreau T Baranger K Dadé S Dallet-Choisy S Guyot N Zani ML Multifaceted roles of human elafin and secretory leukocyte proteinase inhibitor (SLPI), two serine protease inhibitors of the chelonianin family Biochimie 2008 90 284 295 10.1016/j.biochi.2007.09.007 17964057

[B31] McAlhany SJ Ressler SJ Larsen M Tuxhorn JA Yang F Dang TD Rowley DR Promotion of angiogenesis by ps20 in the differential reactive stroma prostate cancer xenograft model Cancer Res 2003 63 5859 5865 14522910

[B32] Berrier AL Yamada KM Cell-matrix adhesion J Cell Physiol 2007 213 565 573 10.1002/jcp.21237 17680633

[B33] Hynes RO Lively JC McCarty JH Taverna D Francis SE Hodivala-Dilke K Xiao Q The diverse roles of integrins and their ligands in angiogenesis Cold Spring Harb Symp Quant Biol 2002 67 143 153 10.1101/sqb.2002.67.143 12858535

[B34] Larsen M Ressler SJ Lu B Gerdes MJ McBride L Dang TD Rowley DR Molecular cloning and expression of ps20 growth inhibitor. A novel WAP-type "four-disulfide core" domain protein expressed in smooth muscle J Biol Chem 1998 273 4574 4584 10.1074/jbc.273.8.4574 9468514

[B35] Ruggiero E Bona R Muratori C Federico M Virological consequences of early events following cell-cell contact between human immunodeficiency virus type 1-infected and uninfected CD4+ cells J Virol 2008 82 7773 7789 10.1128/JVI.00695-08 18508887 PMC2519596

[B36] Blanco J Bosch B Fernández-Figueras MT Barretina J Clotet B Esté JA High level of coreceptor-independent HIV transfer induced by contacts between primary CD4 T cells J Biol Chem 2004 279 51305 51314 10.1074/jbc.M408547200 15371410

[B37] Bosch B Grigorov B Senserrich J Clotet B Darlix JL Muriaux D Este JA A clathrin-dynamin-dependent endocytic pathway for the uptake of HIV-1 by direct T cell-T cell transmission Antiviral Res 2008 80 185 193 10.1016/j.antiviral.2008.06.004 18602423

[B38] Puigdomenech I Massanella M Cabrera C Clotet B Blanco J On the steps of cell-to-cell HIV transmission between CD4 T cells Retrovirology 2009 6 89 10.1186/1742-4690-6-89 19825175 PMC2768678

[B39] Bousso P Robey EA Dynamic behavior of T cells and thymocytes in lymphoid organs as revealed by two-photon microscopy Immunity 2004 21 349 355 10.1016/j.immuni.2004.08.005 15357946

[B40] Celli S Garcia Z Bousso P CD4 T cells integrate signals delivered during successive DC encounters in vivo J Exp Med 2005 202 1271 1278 10.1084/jem.20051018 16275764 PMC2213240

[B41] Martin N Welsch S Jolly C Briggs JA Vaux D Sattentau QJ Virological synapse-mediated spread of human immunodeficiency virus type 1 between T cells is sensitive to entry inhibition J Virol 2010 84 3516 3527 10.1128/JVI.02651-09 20089656 PMC2838118

[B42] Vasiliver-Shamis G Cho MW Hioe CE Dustin ML Human immunodeficiency virus type 1 envelope gp120-induced partial T-cell receptor signaling creates an F-actin-depleted zone in the virological synapse J Virol 2009 83 11341 11355 10.1128/JVI.01440-09 19710135 PMC2772796

[B43] Pais-Correia AM Sachse M Guadagnini S Robbiati V Lasserre R Gessain A Gout O Alcover A Thoulouze MI Biofilm-like extracellular viral assemblies mediate HTLV-1 cell-to-cell transmission at virological synapses Nat Med 2010 16 83 89 10.1038/nm.2065 20023636

[B44] Stewart PL Nemerow GR Cell integrins: commonly used receptors for diverse viral pathogens Trends Microbiol 2007 15 500 507 10.1016/j.tim.2007.10.001 17988871

[B45] Vyakarnam A Eyeson J Teo I Zuckerman M Babaahmady K Schuitemaker H Shaunak S Rostron T Rowland-Jones S Simmons G Clapham P Evidence for a post-entry barrier to R5 HIV-1 infection of CD4 memory T cells AIDS 2001 15 1613 1626 10.1097/00002030-200109070-00003 11546935

[B46] Wu Y Marsh JW Selective transcription and modulation of resting T cell activity by preintegrated HIV DNA Science 2001 293 1503 1506 10.1126/science.1061548 11520990

