# Supplementary figures and images for: WFDC1 expression identifies memory CD4 T- lymphocytes rendered vulnerable to cell-cell HIV-1 transfer by promoting intercellular adhesive junctions
Source: Retrovirology. 2011 May 5;8:29. doi: 10.1186/1742-4690-8-29 (PMC3108927; doi:10.1186/1742-4690-8-29)

Figure 1 supplementary

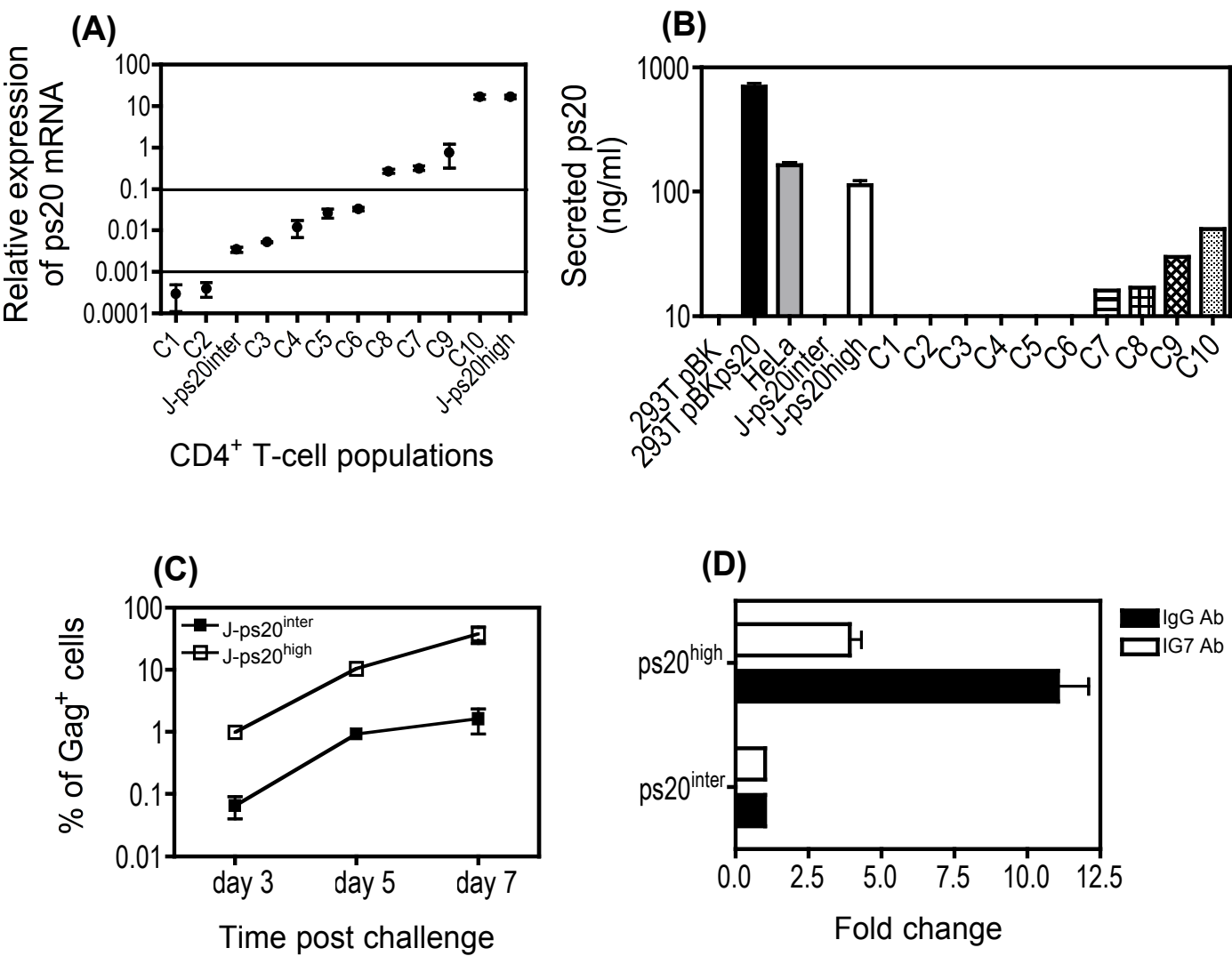

Supplement: Additional file 1 — Figure S1. Jurkat CD4 T cells stably transduced to express full-length human ps20 are more susceptible to cell-free HIV-1 infection. A) The mean relative copy number (RCN) of ps20 mRNA (relative to the HPRT house keeping gene) measured in three biological replicate samples by qRT-PCR; in the empty vector (EV) transduced ps20inter Jurkat cells, WFDC1/ps20 transduced ps20high Jurkat cells, wild-type Jurkat cells as well as a panel of 10 primary CD4+ T-cell clones (C1-C10) is shown. The two horizontal lines are arbitrarily set to define CD4 T-lymphocyte populations as ps20 low (RCN <0.001), ps20 intermediate (RCN 0.001-0.1) and ps20 high (>0.1RCN). (B) Mean secreted ps20 protein levels measured in three replicate samples by a ps20 specific ELISA assay are shown. Positive control was supernatant from the human embryonic kidney cell line, 293T cells transfected with a WFDC1/ps20 encoding construct (pBKps20) and supernatant from ps20 mRNA high HeLa cells; negative control was supernatant from 293T cells transfected with empty vector alone (pBK). Test samples included 48-hour culture supernatant from the WFDC1/ps20 transduced population and the empty vector transduced EV population. (C) 2 × 10^5^ J-ps20inter or J-ps20high cells were challenged with 2.5 ng/1 × 106 cells of the X4-tropic HIV-1 strain, NL4-3. Productive infection was measured by intracellular staining for HIV-1 p24 capsid antigen and the percentage of p24 positive cells determined on days 3, 5 and 7 post-challenge. (D) J-ps20inter or J-ps20high cells were first pre-cultured overnight (16 hrs) in 5 μg/ml of either control mouse IgG1 or the anti-ps20 Ab, IG7, then challenged with NL4-3 for 24 hours (10 ug Gag p24 antigen concentration of virus stock/105 cells). 24 hours later equivalent numbers of cells were trypsinized to remove surface bound virus, washed and cell pellets lysed in PBS with 10% triton-X 100. The amount of Gag p24 antigen was then measured by ELISA and used to assess the fold increase in infectio [file 1742-4690-8-29-S1.PDF]
